# Thinning of Macular Neuroretinal Layers Contributes to Sleep Disorder in Patients With Type 2 Diabetes Without Clinical Evidences of Neuropathy and Retinopathy

**DOI:** 10.3389/fendo.2020.00069

**Published:** 2020-02-28

**Authors:** Fukashi Ishibashi, Mitra Tavakoli

**Affiliations:** ^1^Ishibashi Clinic, Hiroshima, Japan; ^2^University of Exeter Medical School, Exeter, United Kingdom

**Keywords:** inappropriate light exposure, optical coherence tomography, Pittsburgh sleep quality index Japanese version, sleep disorder, thinning of macular neuroretinal layer, type 2 diabetes

## Abstract

**Aims:** To investigate the impact of thinning at individual grids of macular neuroretinal layers, clinical factors, and inadequate light exposure on the specific components of sleep disorder in patients with type 2 diabetes.

**Methods:** One hundred twenty-four patients with type 2 diabetes without clinical evidences of diabetic retinopathy and neuropathy (HbA1c: 8.3%, diabetes duration; 8.7 years) and 54 age- and sex-matched control subjects (HbA1c: 5.6%) underwent detailed clinical, neurological, and ophthalmological examinations. The sleep disorder was assessed by the Pittsburgh Sleep Quality Index Japanese Version (PSQI-J). The temporal structures of daily life were assessed by the Munich Chronotype Questionnaire Japanese Version. The thickness at nine grids defined by the Early Treatment Diabetic Retinopathy Study of nine macular neuroretinal layers was determined by swept-source optical coherence tomography and OCT-Explorer. The associations between the individual components of sleep disorders and the thickness at each grid of macular neuroretinal layers, clinical factors, or the temporal structures of daily life were examined.

**Results:** The prevalence of the sleep disorder, global score, and four individual PSQI-J scores in patients with type 2 diabetes were higher than control subjects. The thickness of two and five grids of two inner retinal layers and four to seven grids of four outer retinal layers in patients with type 2 diabetes was thinner than those in control subjects. The thickness at one to eight grids of four outer retinal layers in type 2 diabetic patients was inversely associated with global score and five individual scores of sleep disorder. The thinning at one to two grids of the inner plexiform layer was related to three high individual scores of sleep disorder. The inappropriate light exposure was associated with the sleep disorder and altered macular neuroretinal layers. The high HbA1c and LDL-cholesterol levels were related to the high global score and two individual scores of sleep disorder, respectively.

**Conclusion:** In patients with type 2 diabetes, the thinning at grids of the inner plexiform layer and outer retinal layers was associated with the high scores of specific components of the sleep disorder. The sleep disorder was also related to hyperglycemia, dyslipidemia, and inappropriate light exposure.

## Introduction

Sleep is essential for health, and people with diabetes are frequently beset with sleep disturbances. According to the American Psychiatric Association, sleep disorder involves problems with the quality, timing, and amount of sleep, which cause problems with functioning and distress during the daytime. However, the concept of sleep disorder in people with diabetes has not been clearly defined and investigated. Sleep disorder is more prevalent in patients with diabetes than people without diabetes (1). Sleep disorder increases the risk of insomnia, hyperglycemia, cardiovascular disease, and cancer (2, 3). In the treatment of diabetes, less attention had been paid to sleep disorder than diet restriction and exercise.

Sleep is controlled by circadian and homeostatic mechanisms (4). The photoentrainment in circadian rhythm is initiated by stimulating intrinsically photosensitive retinal ganglion cells (ipRGCs) with strong blue light (5). The blue light stimulus is transmitted via the suprachiasmatic nucleus to the pineal gland, which secretes melatonin and controls sleep (6). Because ipRGCs receive signals from photoreceptors via the synapse with rod bipolar cells and amacrine cells (7), the altered circadian rhythm might be caused by impaired ipRGCs, photoreceptors, or/and a synapse between them. Although the temporal structures of daily life influence sleep, their impacts on the macular neuroretinal layer thickness had never been reported. Diabetic retinopathy and neuropathy influence the thickness of the macular neuroretinal layers (8, 9) and sleep disorder (10, 11); however, these have not been investigated in detail. Recently, Dumpala et al. (12) reported that the thinning and dysfunction of the outer retina in the small number of patients with type 2 diabetes may contribute to poor sleep behavior. However, they did not find a significant direct association between the thicknesses at the individual grids defined by the Early Treatment Diabetic Retinopathy Study (ETDRS) of outer retinal layers and the components of sleep disorder.

In the current paper, we assessed the impact of the thinning at individual ETDRS grids of all macular neuroretinal layers, clinical factors, and the inadequate light exposure on each component of sleep disorder in type 2 diabetic patients without clinical evidence of diabetic retinopathy and neuropathy.

## Subjects and Methods

### Subjects

One hundred forty-one Japanese patients with type 2 diabetes and 54 sex- and age-matched healthy control subjects were investigated. The exclusion criteria were as follows: older than 55 years, shift worker, restless legs syndrome, sleep apnea, crystalline lens opacity, any grade of diabetic retinopathy, color blindness, best-corrected visual acuity < 1.0, signs of glaucomatous optic discs, and history of ophthalmic surgery.

### Clinical and Laboratory Data

BMI, blood pressure, HbA1c levels, serum creatinine, lipid levels (LDL-cholesterol, HDL-cholesterol, and triglycerides), and urinary creatinine and albumin were measured in all subjects.

### Evaluation of Sleep Disorder and Temporal Structures of Daily Life

The Pittsburgh Sleep Quality Index Japanese Version (PSQI-J) was used to assess the subjective sleep disorder over the past month. PSQI-J contains 18-item self-report questionnaires: these items produce seven components (sleep quality, sleep-onset latency, sleep duration, sleep efficiency, sleep disturbance, use of sleep medications, and daytime dysfunction) that range from 0 (no difficulty) to 3 (severe difficulty). The sum of these component scores yields a global sleep quality (global score). The global score > 5.5 was considered to have sleep disorder (13, 14). The presence of pain during sleep was recorded by PSQI-J. The chronotype and social jetlag were evaluated by Munich Chronotype Questionnaire-Japanese Version based on typical behavior on workdays and work-free days over the past 4 weeks. The chronotype was assessed as midsleep on free days sleep corrected. The temporal structures of daily life were assessed separately for workdays and free days. As the period of various light source exposures, the daily duration of television watching, personal computer operation, mobile phone use, and spent outside under the sunlight was estimated using a personal life behavior table (15).

### Assessment of Neuropathy

All subjects underwent detailed clinical and neurological examinations. Neurological deficits were assessed using the modified neuropathy disability score (16), which evaluates vibration perception, pinprick, temperature perception, and ankle reflexes. The classification for evaluation of neuropathy was based on the Toronto neuropathy consensus (17), which considers a confirmed diabetic peripheral neuropathy as a combination of the presence of abnormal nerve conduction velocity and a symptom, or a sign. As a result, patients with neuropathy disability score > 2 and sensory nerve conduction velocity of sural nerve < 42 m/s were labeled with neuropathy, which has been excluded from this study. Electrophysiology and nerve conduction velocity studies were performed using an electromyography instrument (Neuropak-S1, NIHON KOHDEN, Tokyo, Japan). The motor nerve conduction velocity of the median nerve, the sensory nerve conduction velocity of the sural nerve, and their action potential amplitudes were determined. Skin temperature was maintained above 32°C. The vibration perception threshold was measured at the left medial malleolus using a biothesiometer (Biomedical Instruments, Newbury, OH, USA). Warm and cold perception thresholds at the dorsum of the foot were determined using a thermal stimulator (Intercross-200, Intercross Co., Tokyo, Japan). The coefficient of variation of R-R intervals (CVR-R) was calculated from the R-R intervals of 200 electrocardiogram samples.

### Corneal Confocal Microscopy

All subjects were examined using a Heidelberg Retina Tomograph III *in vivo* corneal confocal microscope with Rostock Corneal Module (Heidelberg Engineering, Heidelberg, Germany) (18). Six high-quality images per subject from Bowman's layer were captured and analyzed to quantify the following corneal nerve fiber morphological parameters: (1) corneal nerve fiber density: total number of major nerve fibers/mm^2^ of corneal tissue; (2) corneal nerve fiber length: total length of all nerve fibers (mm/mm^2^); (3) corneal nerve branch density: number of branches emanating from all major nerve trunks/mm^2^; (4) beading frequency/0.1 mm); and (5) bead size (μm^2^) (19). Except for bead size, all measurements were performed using ImageJ (Texelcraft, Tokyo, Japan).

### Swept-Source Optical Coherence Tomography (SS-OCT) and Automated Segmentation

The OCT images of the right eye were obtained with SS-OCT (DRI OCT Triton, Topcon Corp., Tokyo, Japan) for all subjects. The only high-quality images with an image quality factor of Topcon ≥ 60% and without artifact were employed. For the segmentation of the all macular neuroretinal layers, OCT-Explorer (20) was implemented to automatically identify nine retinal interfaces and assess the following layer thickness: (1) retinal nerve fiber (RNFL), (2) ganglion cell (GCL), (3) inner plexiform (IPL), (4) inner nuclear (INL), (5) outer plexiform (OPL), (6) outer nuclear (ONL), (7) junction of inner and outer segment (OS) of photoreceptor (IS/OS), (8) OS, (9) OS/retinal pigment epithelium (RPE) complex (OPR), and (10) RPE (RPE + Bruch's membrane). The thickness of the central, pericentral, and peripheral rings with diameters of 1, 3, and 6 mm defined by ETDRS was measured. The thickness of the following nine grids of the macular neuroretinal layers and RPE was estimated: central fovea, inner and outer nasal, superior, temporal, and inferior.

### Ophthalmic Examinations

The visual acuity was measured using the international type visual acuity chart (Tsutsumi, Tokyo, Japan). The color blindness was diagnosed by the Ishihara color test. The bilateral fundus images were captured (the field of assessment: 45°) for all subjects at the time of SS-OCT using DRI OCT Triton (Topcon Corp., Tokyo, Japan) without pupil dilatation.

### Statistical Analysis

All statistical analyses were performed using SPSS ver. 19 (SPSS, Chicago, IL, USA), and *p* < 0.05 was considered statistically significant. A *post-hoc* analysis of sample power using Gpower 3.1 (http://gpower.software.informer.com/3.1/) was conducted using a Mann–Whitney test (significance of 0.05) for PSQI-J scores and OCT measures. The present study population provided statistical power ranging from 0.71 to 0.99.

All values are presented as the mean ± standard error of the mean. All data sets were tested for normality using the Shapiro–Wilk test. The differences between controls and type 2 diabetic patients without diabetic neuropathy or between patients with or without statin use were assessed using *t*-test and Mann-Whitney test for normally and non-normally distributed continuous variables and χ^2^ test for categorical variables. Correlations between the PSQI-J scores and clinical factors were assessed with multiple regression analysis. The associations among the PSQI-J scores, neurophysiological tests, corneal nerve fiber measures, thickness at grids of macular neuroretinal layers, clinical factors, and temporal structures of daily life in patients with type 2 diabetes were assessed using Spearman's rank correlation coefficient analysis.

## Results

In order to exclude the potential impact of diabetic neuropathy on sleep disorder and the thickness of the macular neuroretinal layers, 17 patients with confirmed diabetic neuropathy defined by Toronto criteria were excluded, and further analyses were performed using 124 patients without diabetic neuropathy. Hence, the current paper focuses only on type 2 diabetic patients without clinical evidences of diabetic neuropathy and retinopathy.

### Clinical Characteristics

Sex and age were matched between controls and diabetic patients without diabetic neuropathy. BMI, blood pressure, HbA1c levels, LDL-cholesterol, triglycerides, and urinary albumin-to-creatinine ratio in patients with type 2 diabetes were higher, and HDL-cholesterol was lower than those in control subjects (Table 1). LDL-cholesterol in diabetic patients with or without statin use was not significantly different (*p* = 0.171).

**Table 1:** Clinical characteristics, neurophysiological tests, and corneal nerve fiber measures in control subjects and patients with type 2 diabetes without diabetic neuropathy.

	**Control subjects**	**Patients with type 2 diabetes without diabetic neuropathy**	***P*-value**
**CLINICAL DATA**			
Number (Male/Female, M %)	54 (34/20, 63.0)	124 (78/46, 62.9)	NS
Age (years)	48.6 ± 1.2	48.6 ± 0.7	NS
Duration of diabetes (years)	−	8.7 ± 0.7	
Body mass index (BMI, kg/m^2^)	23.3 ± 0.4	27.6 ± 0.5	<0.001
Systolic blood pressure (SBP, mmHg)	127 ± 1.7	132 ± 1.5	0.049
Diastolic blood pressure (DBP, mmHg)	75.6 ± 1.1	79.7 ± 0.9	0.018
No. treated with angiotensin receptor blocker (%)	18.5	27.4	NS
HbA1c (%)	5.6 ± 0.05	8.3 ± 0.17	<0.001
Low density lipoprotein (LDL) cholesterol (mmol/L)	3.02 ± 0.09	3.51 ± 0.08	<0.001
No. treated with statins (%)	14.8	11.2	NS
High density lipoprotein (HDL) cholesterol (mmol/L)	1.76 ± 0.07	1.43 ± 0.04	<0.001
Triglycerides (mmol/L)	1.49 ± 0.19	2.53 ± 0.23	<0.001
Urinary albumin to creatinine ratio (ACR, mg/g creatinine)	9.45 ± 1.4	63.9 ± 19.5	0.001
Estimated glomerular filtration rate (eGFR, ml/min)	84.3 ± 2.5	87.0 ± 1.9	NS
**NEUROPHYSIOLOGICAL TESTS**			
Neuropathy disability score	0.43 ± 0.07	3.84 ± 0.24	<0.001
Motor nerve conduction velocity of median nerve (m/s)	57.9 ± 0.65	53.8 ± 0.38	<0.001
Amplitude of median nerve (mV)	8.72 ± 0.41	7.51 ± 0.30	0.004
Sensory nerve conduction velocity of sural nerve (m/s)	48.5 ± 0.62	47.7 ± 0.42	NS
Amplitude of sural nerve (μV)	15.4 ± 0.94	12.4 ± 0.55	0.004
Vibration perception threshold (μ/120cycles/s)	1.94 ± 0.16	2.94 ± 0.24	0.008
Coefficient of variation of R-R interval (%)	4.05 ± 0.19	3.60 ± 0.13	0.027
Warm perception threshold (W/m^2^)	−468 ± 10.6	−671 ± 74.0	<0.001
Cold perception threshold (W/m^2^)	454 ± 12.1	496 ± 12.7	NS
**CORNEAL NERVE FIBER MEASURES**			
Corneal nerve fiber density (no/mm^2^)	30.9 ± 0.78	22.8 ± 0.42	<0.001
Corneal nerve fiber length (mm/mm^2^)	15.0 ± 0.28	12.1 ± 0.19	<0.001
Corneal nerve branch density (no/mm^2^)	13.5 ± 0.65	11.3 ± 0.31	0.012
Beading frequency (no/0.1mm)	23.5 ± 0.26	20.3 ± 0.16	<0.001
Bead size (μm^2^)	8.18 ± 0.075	10.8 ± 0.092	<0.001

*Data are the mean ± standard error of the mean in control subjects and patients with type 2 diabetes without diabetic neuropathy*.

*P-values are between control subjects and type 2 diabetic patients without neuropathy*.

### Subjective Sleep Disorder and Temporal Structures of Daily Life

The global score, prevalence of sleep disorder, and four individual scores of sleep disorder in patients were significantly higher than controls (Table 2). In patients without statin use, the global score (*p* = 0.016) and sleep duration score (*p* = 0.048) were higher than patients with statin use. Among 124 patients without diabetic neuropathy, 11 (8.9%) patients complained of some pain during sleep. The prevalence of sleep disorder in patients with pain was higher than patients without pain (*p* = 0.043). None of individual sleep disorder scores was different between patients with or without pain (*p* = 0.082–0.380).

**Table 2:** Subjective sleep disorder estimated by Pittsburgh Sleep Quality Index Japanese Version and temporal structures of daily life estimated by Munich Chronotype Questionnaire Japanese Version in control subjects and patients with type 2 diabetes without diabetic neuropathy.

		**Control subjects**	**Patients with type 2 diabetes without diabetic neuropathy**	***P*-value**
**SCORES OF SUBJECTIVE SLEEP DISORDER ESTIMATED BY PSQI-J**				
Global score		3.26 ± 0.22	4.80 ± 0.24	<0.001
Prevalence of sleep disorder (global score: > 5.5%)		3.7	28.2	<0.001
Sleep quality		0.89 ± 0.09	1.20 ± 0.07	0.007
Sleep-onset latency		0.30 ± 0.07	0.68 ± 0.08	0.004
Sleep duration		1.11 ± 0.11	1.25 ± 0.08	NS
Sleep efficiency		0.06 ± 0.031	0.19 ± 0.053	NS
Sleep disturbance		0.67 ± 0.070	0.80 ± 0.049	NS
Use of sleep medications		0	0.17 ± 0.058	0.043
Daytime dysfunction		0.19 ± 0.053	0.52 ± 0.068	0.004
**TEMPORAL STRUCTURES OF DAILY LIFE ESTIMATED BY MCQ-J**				
Television watching time (min/day)	Work day	91.3 ± 7.2	150 ± 11.3	0.003
	Free day	127 ± 10.9	216 ± 15.4	0.001
Personal computer operating time (min/day)	Work day	124 ± 19.3	124 ± 16.1	NS
	Free day	25.9 ± 6.7	26.7 ± 5.5	NS
Mobile phone using time (min/day)	Work day	62.0 ± 10.3	69.5 ± 6.3	NS
	Free day	60.6 ± 9.9	76.0 ± 7.0	NS
Sunlight exposure time (min/day) at outdoor	Work day	96.0 ± 16.0	86.7 ± 12.3	NS
	Free day	135 ± 14.3	85.9 ± 10.6	<0.001
Chronotype (local time)		02:58±(19min)	03:10±(15min)	NS
Social jet-lag (hr)		0.98 ± 0.28	0.73 ± 0.08	NS

*Data are the mean ± standard error of the mean in control subjects and patients with type 2 diabetes without diabetic neuropathy*.

*P-values are between control subjects and type 2 diabetic patients without diabetic neuropathy*.

*MCQ-J, Munich Chronotype Questionnaire-Japanese Version; PSQI-J, Pittsburgh Sleep Quality Index Japanese Version*.

Television watching time on work and the free day was longer, and sunlight exposure time on free day was shorter in patients without diabetic neuropathy than controls (Table 2).

### Neurophysiological Tests and Corneal Nerve Fiber Measures

Neuropathy disability score was higher in patients with type 2 diabetes than controls. All neurophysiological tests except for the sensory nerve conduction velocity of sural nerve and cold perception threshold in patients without diabetic neuropathy were impaired compared with controls. All corneal nerve fiber morphological measures in patients without diabetic neuropathy were altered compared with controls (Table 1).

### Alterations in Macular Neuroretinal Layers and RPE

**Figure 1:**
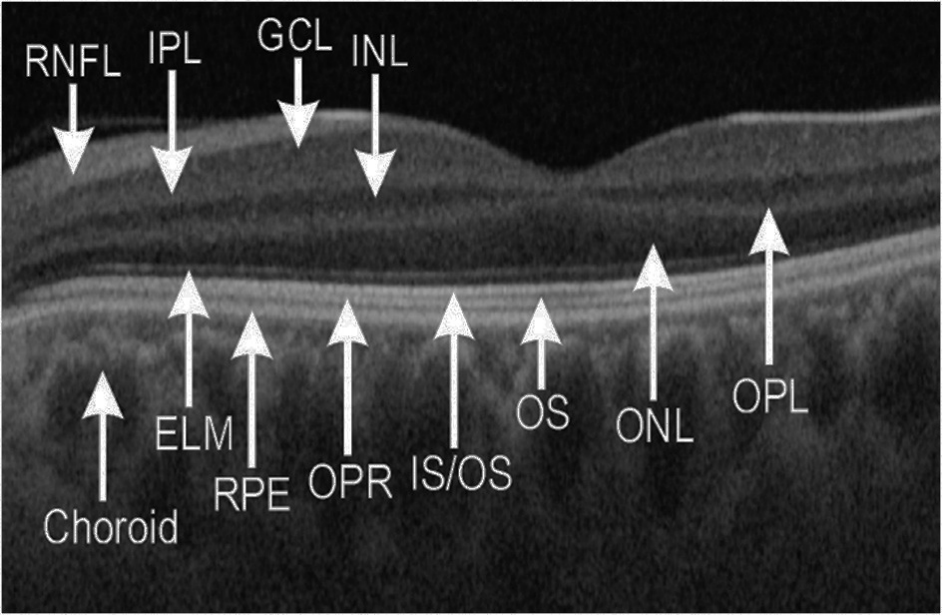
Representative optical coherence tomography (OCT) image of macular neuroretinal layers visualized by swept-source OCT and OCT-Explorer in a healthy control subject. ELM, external limiting membrane; GCL, ganglion cell layer; INL, inner nuclear layer; IPL, inner plexiform layer; IS/OS, junction of inner segment/outer segment of photoreceptor; ONL, outer nuclear layer; OPL, outer plexiform layer; OPR, outer segment of photoreceptor/retinal pigment epithelium complex; OS, outer segment; RNFL, retinal nerve fiber layer; RPE, retinal pigment epithelium.

The representative OCT image of a control subject showing individual macular neuroretinal layers is presented in Figure 1. At the inner retinal layers, the thickness at two grids of GCL and five grids of IPL in patients with type 2 diabetes was thinner than that in control subjects. At the outer retinal layers, the thickness at four ONL grids, seven IS/OS grids, four OS grids, and six OPR grids in patients with type 2 diabetes was thinner than that in control subjects. The thickness at an RPE grid in patients was thinner than that in control subjects (Table 3).

**Table 3:** Thickness at ETDRS grids of macular neuroretinal layers and retinal pigment epithelium in control subjects and patients with type 2 diabetes without diabetic neuropathy.

		**Control subjects**	**Patients with type 2 diabetes without diabetic neuropathy**	***P*-value**
**Thickness at ETDRS grids of macular neuroretinal layers and retinal pigment epithelium (μm)**				
Ganglion cell layer	Inner superior	49.3 ± 0.97	45.8 ± 0.75	0.010
	Inner inferior	45.4 ± 0.98	42.7 ± 0.66	0.036
Inner plexiform layer	Central fovea	24.2 ± 0.65	22.1 ± 0.32	0.009
	Inner nasal	41.9 ± 0.77	38.7 ± 0.37	0.022
	Inner superior	37.8 ± 0.65	35.5 ± 0.39	0.003
	Outer superior	40.6 ± 0.47	38.5 ± 0.34	0.039
	Outer inferior	40.7 ± 0.49	39.3 ± 0.33	0.012
Outer nuclear layer	Inner superior	89.9 ± 1.7	85.1 ± 1.0	0.008
	Inner temporal	93.5 ± 1.3	89.5 ± 0.97	0.010
	Outer superior	79.7 ± 1.0	76.8 ± 0.66	0.031
	Outer temporal	73.9 ± 0.73	70.7 ± 0.61	0.002
IS/OS	Central fovea	15.6 ± 0.11	15.0 ± 0.11	<0.001
	Inner nasal	13.6 ± 0.12	12.9 ± 0.10	<0.001
	Inner superior	13.9 ± 0.10	13.3 ± 0.09	<0.001
	Inner temporal	13.8 ± 0.10	13.1 ± 0.10	<0.001
	Outer nasal	12.1 ± 0.17	11.3 ± 0.15	0.001
	Outer superior	13.1 ± 0.10	12.5 ± 0.11	<0.001
	Outer inferior	13.0 ± 0.10	12.5 ± 0.11	0.002
Outer segment	Inner nasal	14.2 ± 0.08	13.9 ± 0.09	0.040
	Inner temporal	14.3 ± 0.08	14.0 ± 0.09	0.049
	Outer nasal	14.0 ± 0.08	13.5 ± 0.13	0.002
	Outer temporal	14.8 ± 0.15	14.2 ± 0.10	0.001
OPR	Central fovea	15.5 ± 0.08	14.9 ± 0.14	0.002
	Inner nasal	14.6 ± 0.07	14.2 ± 0.13	0.049
	Inner temporal	14.7 ± 0.06	14.2 ± 0.13	0.004
	Inner inferior	14.8 ± 0.07	14.3 ± 0.13	0.022
	Outer temporal	14.1 ± 0.09	13.7 ± 0.13	0.019
	Outer inferior	14.4 ± 0.09	13.9 ± 0.13	0.043
Retinal pigment epithelium	Outer superior	18.3 ± 0.10	17.7 ± 0.13	0.022

*Data are the mean ± standard error of the mean in control subjects and patients with type 2 diabetes without diabetic neuropathy*.

*P-values are between control subjects and type 2 diabetic patients without diabetic neuropathy*.

*ETDRS, Early Treatment Diabetic Retinopathy Study; IS/OS, junction of inner and outer segment of photoreceptor;OPR, outer segment of photoreceptor/retinal pigment epithelium complex*.

### Associations Among PSQI-J Scores, Clinical Characteristics, Thickness of Macular Neuroretinal Layers, Neurophysiological Tests, Corneal Nerve Fiber Measures, and Temporal Structures of Daily Life in Patients With Type 2 Diabetes Without Diabetic Neuropathy

HbA1c levels were positively associated with the global score (β = 0.187, *p* = 0.037) and the scores of use of sleep medications (β = 0.196, *p* = 0.029) and daytime dysfunction (β = 0.312, *p* < 0.001). Serum LDL-cholesterol levels were positively associated with the global score (β = 0.177, *p* = 0.049) and the scores of sleep quality (β = 0.250, *p* = 0.005) and daytime dysfunction (β = 0.315, *p* < 0.001). Unexpectedly, age and BMI were not related to the scores of sleep disorder.

The global score was negatively associated with an ONL grid thickness. The sleep quality score was negatively related to the thickness at a grid of IS/OS, two grids of OS, eight grids of OPR, and median nerve amplitude. The sleep efficiency score was negatively related to a thickness at two IPL grids, a grid of ONL and IS/OS, and five grids of OPR and CVR-R. The sleep disturbance score was negatively associated with the thickness at an IPL grid and three OPR grids. The use of sleep medication score was negatively associated with the thickness at a grid of IPL and INL, four ONL grids, and beading frequency. The daytime dysfunction score was negatively linked to the thickness at seven ONL grids and positively linked to bead size (Table 4).

**Table 4:** Linear regression between components of Pittsburgh Sleep Quality Index Japanese Version and the thickness at grids defined by Early Treatment Diabetic Retinopathy Study of macular neuroretinal layers, neurophysiological tests or corneal nerve fiber measures in patients with type 2 diabetes without diabetic neuropathy.

	**Scores of Pittsburgh Sleep Quality Index Japanese Version**															
	**Global**		**Sleep quality**		**Sleep-onset latency**		**Sleep duration**		**Sleep efficiency**		**Sleep disturbance**		**Sleep medications**		**Daytime dysfunction**	
	***r***	***p***	***r***	***p***	***r***	***p***	***r***	***p***	***r***	***p***	***r***	***p***	***r***	***p***	***r***	***p***
**THICKNESS OF MNL**																
**Inner plexiform**																
Inner superior	−0.074	0.416	−0.137	0.129	0.053	0.555	0.055	0.544	−0.178	**0.048**	−0.180	**0.046**	−0.107	0.236	−0.109	0.230
Inner temporal	−0.091	0.313	−0.119	0.188	0.066	0.469	−0.075	0.411	−0.226	**0.012**	−0.125	0.167	−0.029	0.747	−0.055	0.547
Outer nasal	−0.058	0.525	−0.116	0.201	−0.041	0.653	0.116	0.199	0.049	0.588	0.055	0.546	−0.212	**0.018**	−0.092	0.310
**Inner nuclear**																
Outer temporal	−0.006	0.950	0.067	0.462	0.007	0.937	0.132	0.143	0.035	0.697	−0.032	0.722	−0.194	**0.031**	0.031	0.729
**Outer nuclear**																
Central fovea	−0.082	0.362	−0.072	0.430	−0.012	0.893	0.132	0.143	−0.069	0.445	0.005	0.956	−0.296	**0.001**	−0.193	**0.032**
Inner nasal	−0.169	0.060	−0.037	0.684	−0.041	0.653	−0.057	0.528	−0.089	0.328	0.021	0.816	−0.192	**0.033**	−0.297	**0.001**
Inner superior	−0.156	0.083	−0.115	0.201	−0.085	0.345	0.030	0.742	0.000	1.000	0.024	0.795	−0.281	**0.002**	−0.268	**0.003**
Inner temporal	−0.189	**0.037**	−0.101	0.265	−0.118	0.192	0.002	0.978	−0.006	0.948	−0.063	0.486	−0.148	0.101	−0.086	0.341
Inner inferior	−0.098	0.277	−0.064	0.482	0.012	0.894	0.049	0.590	−0.184	**0.044**	0.013	0.888	−0.140	0.120	−0.207	**0.021**
Outer nasal	−0.126	0.162	−0.008	0.934	−0.055	0.541	−0.061	0.501	0.016	0.857	−0.002	0.980	−0.147	0.102	−0.351	**<0.001**
Outer superior	−0.141	0.120	−0.041	0.649	−0.136	0.132	−0.017	0.853	−0.037	0.683	0.017	0.847	−0.259	**0.004**	−0.192	**0.033**
Outer inferior	−0.109	0.229	−0.050	0.581	−0.036	0.691	0.026	0.778	−0.147	0.104	−0.024	0.793	−0.165	0.068	−0.284	**0.001**
**IS/OS**																
Outer nasal	−0.083	0.357	−0.243	**0.006**	−0.036	0.694	−0.106	0.242	−0.193	**0.032**	0.028	0.755	0.124	0.170	−0.050	0.579
**Outer segment**																
Outer nasal	−0.075	0.406	−0.182	**0.043**	−0.007	0.942	−0.058	0.524	−0.111	0.218	0.082	0.363	0.003	0.977	−0.035	0.700
Outer inferior	−0.049	0.587	−0.195	**0.030**	0.012	0.894	−0.042	0.639	−0.077	0.397	−0.008	0.926	0.050	0.583	−0.022	0.807
**OPR**																
Inner nasal	−0.044	0.630	−0.178	**0.049**	0.049	0.592	−0.044	0.630	−0.207	**0.021**	0.028	0.759	0.000	0.997	−0.040	0.659
Inner superior	−0.037	0.684	−0.183	**0.042**	0.031	0.729	0.009	0.918	−0.129	0.152	−0.126	0.162	0.046	0.615	−0.021	0.815
Inner temporal	−0.097	0.284	−0.261	**0.003**	0.069	0.446	0.074	0.412	−0.213	**0.018**	−0.186	**0.039**	−0.031	0.733	−0.102	0.260
Inner inferior	−0.106	0.243	−0.277	**0.002**	0.059	0.516	0.052	0.567	−0.124	0.170	−0.207	**0.021**	−0.047	0.607	−0.147	0.103
Outer nasal	−0.130	0.152	−0.193	**0.034**	0.038	0.677	0.017	0.815	−0.155	0.086	0.028	0.755	−0.112	0.214	−0.163	0.070
Outer superior	−0.129	0.154	−0.209	**0.020**	0.050	0.580	0.062	0.495	−0.235	**0.009**	−0.138	0.125	−0.087	0.338	−0.064	0.477
Outer temporal	−0.105	0.244	−0.209	**0.020**	0.009	0.917	0.119	0.190	−0.220	**0.014**	−0.179	**0.048**	−0.045	0.623	−0.066	0.470
Outer inferior	−0.116	0.200	−0.184	**0.041**	−0.025	0.779	0.085	0.348	−0.202	**0.024**	−0.065	0.471	−0.069	0.448	−0.149	0.098
**Neurophysiological test**																
Amp of median N	0.029	0.753	−0.200	**0.026**	−0.110	0.222	0.053	0.559	−0.103	0.256	−0.054	0.548	0.130	0.152	−0.042	0.646
CVR-R	−0.008	0.925	−0.119	0.189	−0.008	0.929	0.009	0.920	−0.227	**0.011**	−0.107	0.236	0.053	0.560	−0.016	0.863
**CNF measures**																
BF	−0.139	0.123	−0.081	0.374	−0.122	0.177	0.012	0.892	0.146	0.106	−0.033	0.716	−0.185	**0.040**	−0.131	0.148
Bead size	0.166	0.066	0.090	0.320	−0.001	0.990	−0.028	0.762	−0.058	0.525	0.154	0.087	0.162	0.073	0.248	**0.006**

*Bold p-value reveals p < 0.05*.

*Amp, amplitude; BF, beading frequency; CNF; corneal nerve fiber; CVR-R, coefficient of variation of R-R interval; IS/OS, junction of inner and outer segment of photoreceptor; MNL, macular neuroretinal layer; N, nerve; OPR, outer segment of photoreceptor/retinal pigment epithelium complex*.

Age was negatively related to the thickness at a grid of RNFL, GCL, and IPL, while it was positively related to the thickness at one to three grids of IS/OS, OS, and RPE. BMI was positively related to the thickness at one to three grids of GCL, INL, and OPL. HbA1c levels were positively related to the thickness at one to three grids of RNFL, GCL, INL, and RPE, and negatively related to the thickness at one to seven grids of IS/OS, OS, and OPR. The LDL-cholesterol levels were positively related to the thickness at one to three grids of three inner retinal layers and negatively related to the thickness at one to three grids of four outer retinal layers. HDL-cholesterol levels were positively associated with the thickness at one to five grids of IPL, ONL, and IS/OS. The triglycerides were negatively related to the thickness at one to four grids of three outer retinal layers, while positively to the thickness at four RPE grids (Table 5).

**Table 5:** Linear regression between clinical factors and thickness at grids defined by Early Treatment Diabetic Retinopathy Study of macular neuroretinal layers and RPE in patients with type 2 diabetes without diabetic neuropathy.

**Grids of macular neuroretinal layers and RPE**	**Clinical factors in patients with type 2 diabetes without diabetic neuropathy**											
	**Age**		**BMI**		**HbA1c**		**LDL cholesterol**		**HDL cholesterol**		**Triglycerides**	
	***r***	***p***	***r***	***p***	***r***	***p***	***r***	***p***	***r***	***p***	***r***	***p***
**RNF**												
Central fovea	−0.244	**0.006**	0.081	0.373	0.141	0.118	−0.062	0.494	−0.107	0.269	0.035	0.697
Inner temporal	0.100	0.270	−0.037	0.687	0.155	0.086	0.202	**0.024**	−0.102	0.259	0.114	0.209
Inner inferior	0.004	0.967	0.115	0.202	0.255	**0.004**	0.065	0.470	−0.170	0.059	0.128	0.157
**Ganglion cell**												
Central fovea	−0.181	**0.044**	0.048	0.595	0.107	0.239	0.045	0.618	−0.083	0.357	0.065	0.473
Inner temporal	0.085	0.351	0.068	0.452	0.083	0.357	0.294	**0.001**	0.016	0.862	0.056	0.539
Inner inferior	−0.005	0.956	0.020	0.830	0.184	**0.041**	0.171	0.058	−0.076	0.401	0.058	0.522
Outer temporal	0.003	0.978	0.185	**0.040**	0.026	0.776	0.157	0.081	−0.011	0.900	0.040	0.656
**Inner plexiform**												
Inner nasal	0.031	0.730	−0.091	0.313	−0.084	0.354	−0.034	0.705	0.178	**0.048**	−0.176	0.050
Inner temporal	−0.180	**0.046**	−0.015	0.871	0.124	0.172	−0.057	0.530	−0.075	0.409	−0.069	0.449
**Inner nuclear**												
Inner nasal	0.063	0.490	0.185	**0.039**	0.170	0.059	0.149	0.098	0.058	0.520	−0.071	0.431
Inner temporal	0.050	0.585	0.022	0.808	0.253	**0.005**	0.206	**0.022**	−0.034	0.708	−0.045	0.621
Inner inferior	−0.001	0.993	0.124	0.169	0.318	**<0.001**	0.189	**0.035**	−0.101	0.262	0.076	0.404
Outer temporal	−0.007	0.937	0.165	0.067	0.066	0.466	0.225	**0.012**	−0.005	0.955	−0.030	0.737
Outer inferior	−0.106	0.240	0.214	**0.017**	0.111	0.220	0.155	0.087	−0.028	0.755	0.010	0.910
**Outer plexiform**												
Inner superior	−0.141	0.118	0.231	**0.010**	0.038	0.678	0.106	0.243	0.117	0.196	−0.170	0.059
Outer superior	−0.047	0.603	0.291	**0.001**	0.013	0.883	0.042	0.640	0.122	0.178	−0.185	**0.040**
Outer inferior	−0.025	0.782	0.196	**0.029**	−0.051	0.575	0.031	0.729	−0.006	0.946	−0.021	0.817
**Outer nuclear**												
Central fovea	0.006	0.943	0.048	0.597	0.078	0.390	−0.194	**0.031**	−0.057	0.529	0.016	0.863
Inner nasal	0.023	0.801	−0.059	0.515	−0.002	0.986	−0.179	**0.047**	−0.035	0.697	−0.036	0.689
Inner superior	0.168	0.063	−0.136	0.131	−0.058	0.522	−0.295	**0.001**	−0.018	0.845	−0.002	0.982
Inner temporal	0.148	0.102	−0.003	0.972	−0.170	0.059	−0.121	0.182	0.150	0.096	−0.217	**0.016**
Outer superior	0.141	0.118	−0.060	0.508	−0.053	0.561	−0.226	**0.012**	0.059	0.517	−0.059	0.515
Outer temporal	0.111	0.222	−0.044	0.629	−0.118	0.193	−0.153	0.090	0.191	**0.034**	−0.207	**0.021**
Outer inferior	0.107	0.236	−0.078	0.390	−0.115	0.203	−0.150	0.095	0.168	0.062	−0.201	**0.025**
**IS/OS**												
Central fovea	0.102	0.259	−0.035	0.699	−0.115	0.202	−0.033	0.719	0.189	**0.036**	−0.139	0.123
Inner nasal	0.066	0.468	−0.045	0.621	−0.183	**0.042**	−0.067	0.457	0.142	0.115	−0.194	**0.031**
Inner superior	0.056	0.535	−0.043	0.632	−0.167	0.064	−0.075	0.409	0.244	**0.006**	−0.191	**0.034**
Inner temporal	0.067	0.463	−0.024	0.795	−0.215	**0.017**	−0.074	0.416	0.156	0.084	−0.159	0.078
Inner inferior	0.025	0.779	−0.040	0.659	−0.235	**0.009**	0.010	0.914	0.191	**0.034**	−0.180	**0.046**
Outer nasal	0.094	0.299	−0.015	0.865	−0.198	**0.028**	−0.287	**0.001**	0.042	0.646	−0.073	0.418
Outer superior	0.196	**0.029**	−0.078	0.389	−0.272	**0.002**	−0.113	0.212	0.278	**0.002**	−0.186	**0.039**
Outer temporal	0.249	**0.005**	−0.062	0.495	−0.222	**0.013**	−0.084	0.351	0.095	0.292	−0.047	0.608
Outer inferior	0.200	**0.026**	−0.085	0.349	−0.308	**0.001**	−0.099	0.272	0.215	**0.016**	−0.132	0.143
**Outer segment**												
Inner inferior	0.024	0.793	0.038	0.674	−0.183	**0.042**	0.047	0.607	0.047	0.608	−0.055	0.541
Outer nasal	−0.043	0.639	−0.019	0.836	−0.105	0.246	−0.185	**0.040**	−0.055	0.544	−0.005	0.957
Outer temporal	0.178	**0.048**	−0.071	0.433	−0.295	**0.001**	−0.099	0.272	0.067	0.463	−0.052	0.570
Outer inferior	0.089	0.324	0.006	0.951	−0.195	**0.030**	−0.086	0.344	0.100	0.268	−0.031	0.729
**OPR**												
Inner temporal	0.025	0.780	−0.055	0.544	−0.200	**0.026**	−0.140	0.121	0.063	0.485	−0.137	0.130
Outer nasal	0.007	0.938	0.022	0.806	−0.107	0.236	−0.251	**0.005**	0.005	0.960	−0.117	0.195
**RPE**												
Central fovea	0.192	**0.032**	−0.069	0.449	0.032	0.724	0.004	0.969	0.018	0.840	−0.021	0.815
Outer nasal	0.013	0.885	0.010	0.916	0.149	0.100	0.140	0.120	−0.075	0.405	0.218	**0.015**
Outer superior	0.009	0.918	−0.016	0.862	0.206	**0.022**	0.065	0.474	−0.156	0.083	0.255	**0.004**
Outer temporal	−0.050	0.578	−0.071	0.430	0.214	**0.017**	0.029	0.750	−0.146	0.105	0.280	**0.002**
Outer inferior	−0.024	0.789	0.020	0.830	0.187	**0.037**	0.150	0.096	−0.097	0.284	0.214	**0.017**

*Bold p-value reveals p < 0.05*.

*BMI, body mass index; HDL, high density lipoprotein; IS/OS, junction of inner and outer segment of photoreceptor; LDL, low density lipoprotein; OPR, outer segment of photoreceptor/retinal pigment epithelium complex; RNF, retinal nerve fiber; RPE, retinal pigment epithelium + Bruch's membrane complex*.

The television watching time on workday was positively associated with the global score and three individual sleep disorder scores and negatively associated with the thickness at a grid of three macular neuroretinal layers. That on free day was negatively related to the thickness at two OPL grids. The time of personal computer operation on workday was negatively linked to the thickness at a GCL and four OPL grids. That on free day was positively associated with the global score and four individual scores of sleep disorder and negatively associated with the thickness at a GCL and OPL grid. The mobile phone use on workday was negatively associated with the thickness at an IS/OS grid. The sunlight exposure on workday was negatively related to the thickness at a GCL grid and six RPE grids, and that on free day was negatively associated with the daytime dysfunction score (Table 6).

**Table 6:** Linear regression between temporal structures of daily life and components of Pittsburgh Sleep Quality Index Japanese Version or the thickness at grids defined by Early Treatment Diabetic Retinopathy Study of macular neuroretinal layers and RPE in patients with type 2 diabetes without diabetic neuropathy.

	**Temporal structures of daily life**															
	**Television watching time**				**PC operating time**				**Mobile phone using time**				**Sunlight exposure time at outside**			
	**Work day**		**Free day**		**Work day**		**Free day**		**Work day**		**Free day**		**Work day**		**Free day**	
	***r***	***p***	***r***	***p***	***r***	***p***	***r***	***p***	***r***	***p***	***r***	***p***	***r***	***p***	***r***	***p***
**PSQI-J Score**																
Global	0.191	**0.033**	0.038	0.677	−0.083	0.358	0.267	**0.003**	0.004	0.965	−0.018	0.845	0.022	0.804	−0.038	0.675
Sleep quality	0.038	0.677	−0.037	0.687	0.100	0.267	0.299	**0.001**	0.077	0.395	0.043	0.637	0.023	0.801	−0.039	0.669
Sleep-onset latency	0.215	**0.017**	0.065	0.471	−0.122	0.178	0.155	0.086	−0.061	0.502	0.001	0.994	0.074	0.413	−0.082	0.368
Sleep duration	0.227	**0.011**	0.109	0.228	0.020	0.822	0.123	0.173	0.059	0.515	−0.008	0.931	−0.027	0.768	0.078	0.388
Sleep efficiency	0.216	**0.016**	−0.048	0.596	0.001	0.987	0.181	**0.045**	0.064	0.478	0.101	0.264	0.057	0.530	0.176	0.051
Sleep disturbance	−0.064	0.478	0.016	0.862	0.053	0.560	0.279	**0.002**	−0.065	0.472	−0.054	0.548	0.149	0.099	0.085	0.346
Sleep medication use	0.022	0.806	−0.136	0.133	−0.067	0.462	0.241	**0.007**	−0.161	0.074	0.065	0.476	−0.038	0.676	0.057	0.528
Daytime dysfunction	0.097	0.283	−0.015	0.872	−0.035	0.700	0.048	0.599	−0.058	0.524	−0.105	0.248	0.046	0.612	−0.178	**0.048**
**Thickness of macular neuroretina and RPE**																
RNFL Inner inferior	−0.197	**0.028**	−0.039	0.664	0.142	0.114	−0.021	0.819	−0.062	0.491	−0.094	0.302	0.119	0.190	0.076	0.403
GCL Inner temporal	−0.119	0.189	−0.050	0.585	−0.027	0.767	0.040	0.662	0.005	0.959	0.053	0.562	−0.186	**0.039**	−0.115	0.204
Outer nasal	0.149	0.099	0.161	0.075	−0.184	**0.041**	−0.190	**0.034**	−0.005	0.959	0.052	0.570	0.034	0.706	−0.059	0.515
INL Inner temporal	−0.194	**0.030**	−0.153	0.089	0.033	0.720	0.023	0.800	0.044	0.630	0.022	0.805	−0.061	0.501	−0.076	0.404
OPL Central fovea	0.038	0.674	−0.053	0.555	−0.181	**0.047**	−0.197	**0.028**	0.147	0.103	0.014	0.877	−0.144	0.110	−0.123	0.172
Inner temporal	−0.252	**0.005**	−0.243	**0.007**	−0.125	0.166	−0.055	0.542	0.015	0.866	−0.101	0.265	0.048	0.594	−0.161	0.073
Inner inferior	−0.004	0.964	−0.075	0.407	−0.211	**0.019**	−0.031	0.733	0.087	0.336	−0.108	0.232	−0.027	0.763	−0.066	0.466
Outer temporal	−0.100	0.267	−0.182	**0.043**	−0.183	**0.045**	−0.091	0.312	0.104	0.251	−0.061	0.500	0.080	0.377	−0.122	0.179
Outer inferior	0.032	0.726	−0.063	0.484	−0.242	**0.007**	−0.062	0.492	0.111	0.219	−0.105	0.248	−0.065	0.475	−0.153	0.091
IS/OS Inner nasal	−0.028	0.755	−0.065	0.473	−0.027	0.769	0.111	0.218	−0.190	**0.035**	−0.069	0.447	0.003	0.975	−0.088	0.330
OS Outer superior	−0.004	0.965	0.037	0.684	−0.126	0.165	0.071	0.432	−0.040	0.659	−0.032	0.723	−0.182	**0.043**	−0.075	0.406
RPE Inner nasal	0.050	0.579	0.069	0.449	−0.076	0.404	−0.063	0.487	−0.122	0.176	−0.096	0.290	−0.220	**0.014**	−0.113	0.213
Inner superior	0.022	0.804	0.083	0.361	−0.055	0.547	−0.051	0.575	−0.137	0.130	−0.097	0.282	−0.185	**0.039**	−0.124	0.171
Outer nasal	0.009	0.918	0.132	0.144	0.089	0.327	0.051	0.572	−0.109	0.229	−0.022	0.810	−0.295	**0.001**	−0.055	0.547
Outer superior	0.019	0.832	0.106	0.243	−0.056	0.539	−0.035	0.703	−0.102	0.261	−0.028	0.757	−0.245	**0.006**	−0.067	0.461
Outer inferior	0.021	0.813	0.110	0.226	−0.053	0.558	−0.054	0.554	−0.035	0.702	0.007	0.942	−0.222	**0.013**	−0.059	0.516

*Bold p-value reveals p < 0.05*.

*GCL, ganglion cell layer; INL, inner nuclear layer; IS/OS, junction of inner and outer segment of photoreceptor; OPL, outer plexiform layer; PC, personal computer; PSQI-J, Pittsburgh Sleep Quality Index Japanese Version; RNFL, retinal nerve fiber layer; RPE, retinal pigment epithelium + Bruch's membrane complex*.

The chronotype was positively associated with the global score (*r* = 0.202, *p* = 0.024), latency of sleep-onset score (*r* = 0.228, *p* = 0.011), and the thickness at two INL grids (*r* = 0.183, 0.210, *p* = 0.042, 0.019). The social jetlag was positively related to the daytime dysfunction score (*r* = 0.204, *p* = 0.023) and negatively related to the thickness at three ONL grids (*r* = −0.196 to −0.230, *p* = 0.029–0.010).

## Discussion

Sleep disorder in people with diabetes has important clinical implications including impaired daytime functioning, poor glucose regulation, and poor quality of life (21). Hence, the assessment of the sleep disorder is essential for the effective management of diabetes (22). However, sleep disorder has not been investigated in detail in patients with diabetes.

The current paper assessed the detailed association between altered neuroretinal structures with the components of the sleep disorder in a cohort of patients with type 2 diabetes without clinical evidences of retinopathy and neuropathy. The sleep disorder was evaluated using the validated PSQI-J. The results revealed the higher prevalence, global score, and four individual scores of the sleep disorder in diabetic patients than controls. The OCT results confirmed the altered neuroretinal structures in diabetic patients compared to controls, and the thinning in outer retinal layers was more marked than that in inner retinal layers. In *db/db* mice, a murine model of spontaneous type 2 diabetes, and other diabetic animal models, the degeneration of neuroretina was found using OCT before the development of diabetic retinopathy (23). The mechanisms of neuroretinal degeneration by diabetes remain to be fully elucidated. However, oxidative stress, extracellular glutamate accumulation, and insufficient retinal production of neuroprotective factors such as pigment epithelial-derived factor and interstitial retinol-binding protein may play a role (24). Photoreceptors contain most of the mitochondria found in the retina and are the major site of superoxide generation in diabetes (25). These results are compatible with the more marked thinning of outer retina than that of inner retina.

Sleep is regulated by homeostatic and circadian mechanisms (4). In the homeostatic mechanism, prolonged wakefulness increases sleep drive, whereas the circadian oscillator partitions sleep within the day and night cycle. In the modern post-industrial society, people are always exposed to light except during sleep and continuous light exposure disturbs sleep, predominantly by modulating the circadian rhythm in a photoentrainment. In the eye, ipRGCs are responsible for conveying retinal light stimuli to suprachiasmatic nucleus (6) and hence setting the photoentrainment of circadian rhythm, and finally, control the melatonin secretion from pineal gland for regulating sleep (26). Because the signal from the ipRGCs is modulated by inputs from the photoreceptors via the synapses with bipolar and amacrine cells (27), the function and morphology of ipRGCs, cone-rod, and synapses between them should be evaluated separately for exploring the causes of the sleep disorder evoked by compromised photoentrainment. Because the ipRGCs occupy only 0.2% of total ganglion cell population at the retina (5), OCT cannot identify ipRGCs. The studies in blind people due to photoreceptor dysfunction (28) and pupillary light reflex using chromatic light (6) evidenced the pivotal role of a small number of ipRGCs in non-visual light sensing and sleep regulation. However, we did not have direct evidence suggesting the causal role of impaired ipRGC function in sleep disorder of diabetic patients. As IPL is composed of the network between ipRGCs and amacrine or bipolar cells, the thinning of IPL potentially contributes to the impairment of ipRGC function. The thickness at five IPL grids in diabetic patients was thinner than controls, suggesting that the thinning of the IPL plays a possible role in high scores of sleep efficiency, sleep disturbance, and the use of sleep medications due to potentially compromised photoentrainment by disrupting the network between ipRGCs and photoreceptors.

The thickness of four ONL grids in patients with diabetes was thinner than control subjects and was inversely related to the global score and scores of sleep efficiency, sleep medication use, and daytime dysfunction. ONL defined by OCT-Explorer (20) contains nuclei and IS of photoreceptor where mitochondria are abundant. Seventy percent of retinal mitochondria locate in photoreceptors, and in diabetes, photoreceptor cells are a major contributor to diabetes-induced oxidative stress and local inflammation in the retina (25). Therefore, the impaired photoreceptor function by the thinning of ONL potentially compromised photoentrainment, resulting in sleep disorder.

OPR plays an important role for the clearance of shed outer segment discs and the maintenance of pH, ion concentration, and fluid balance in subretinal space. The thickness of six OPR grids in diabetic patients was thinner than that in control subjects. It is plausible that the thinning of OPR had robust inverse relationship to the scores of sleep quality (integrated aspects of sleep process), sleep efficiency, and sleep disturbance of sleep disorder.

Recently, Dumpala et al. (12) reported the outer retinal thinning and dysfunction in a small number of patients with type 2 diabetes and inferred that outer retinal structure and function deficits contribute to the sleep disruption in type 2 diabetes. However, they determined the thickness of total outer retina and did not directly associate the individual components of sleep disorder with the thickness at ETDRS grids of each outer retinal layers.

The detailed association between sleep disorder and the thinning of the macular neuroretinal layers in patients with type 2 diabetes had never been investigated. In the present study, we determined the thickness at nine grids defined by ETDRS of nine macular neuroretinal layers and RPE. The thickness at one to seven grids of six macular neuroretinal layers and RPE in patients without diabetic neuropathy was significantly thinner than those in controls. However, the influence of diabetes on the thickness of the macular neuroretinal layers had not been consistent (8, 29), because OCT image quality depends on the instrument reliability and measurement variability (30). SS-OCT uses 1 μ wavelength light source, allowing deeper penetration into the photoreceptors and RPE (31). Because our SS-OCT cannot determine the individual thickness of the macular neuroretinal layers, we assessed it by processing SS-OCT images using OCT-Explorer (20), which enabled us to measure the thickness as thin as 10 μm. The CV of five measurements over 6 months in nine ETDRS grids of nine macular neuroretinal layers and RPE in 10 control subjects were <5%, indicating good repeatability.

The sleep quality score was inversely related to the thickness of photoreceptors. HbA1c levels, LDL-cholesterol, and triglycerides negatively and HDL-cholesterol positively influenced the thickness of photoreceptors. Therefore, for improving sleep quality, hyperglycemia and dyslipidemia should be controlled. The previous cross-sectional studies revealed that sleep disorder contributed to hyperglycemia and dyslipidemia, while no prospective study indicating that the treatment of hyperglycemia or dyslipidemia improves sleep quality was found. Therefore, the benefit of treating hyperglycemia and dyslipidemia on sleep quality is tentative at the present time. The ONL thickness had a close negative relationship with the use of sleep medications and daytime dysfunction and had potentially negative association with LDL-cholesterol and triglycerides and positive association with HDL-cholesterol. Correcting dyslipidemia can reduce sleep medications and improve daytime dysfunction.

Aging had potentially negative association with the thickness of inner retinal layers and positive association with the thickness of outer retinal layers and RPE (32, 33). RPE enlarges by aging due to the multinucleation (33). In a healthy population (34), aging is associated with the photoreceptor thinning and the thickening of RPE. The much older mean age (70.6 years) of the above study population than ours (48.6 years) might produce the difference. In the present study, BMI had a potentially positive influence on the thickness of inner retinal layers and OPL, while in the previous studies (34, 35), BMI does not affect the thickness of the macular neuroretinal layers. We did not have plausible explanation for the difference. The positive relationship between the INL thickness and HbA1c levels might be due to the hyperglycemia-induced swelling of Müller cells (36). The HbA1c levels negatively affected the thickness of photoreceptors and positively affected that of the inner retinal layers and RPE. Because the photoreceptors are a dominant source of retinal oxidative stress caused by hyperglycemia (25), diabetes may degenerate the photoreceptors. In the diabetic and hypercholesterolemic pig model, the blood-retinal barrier is impaired, and plasma components leak into RPE (37), making RPE edematous. In hypercholesterolemic rabbits, ONL was degenerated (38). These histological changes in animal models were compatible with the current findings that serum LDL-cholesterol levels negatively influenced the ONL thickness, contributing to the use of sleep medication and daytime dysfunction. The serum LDL-cholesterol levels were positively associated with the global score and two individual PSQI-J scores. The high total and LDL-cholesterol levels (39, 40) were associated with sleep disorder. We could not exclude the causal role of sleep disorder in inducing hypercholesterolemia.

The inappropriate light exposure may be associated with sleep disorder and reduced thickness of the macular neuroretinal layers. We estimated the duration of television watching, PC operation, mobile phone use, and sunlight exposure but did not measure the intensity of light during these periods. Therefore, we could not determine the quantitative association between amount of these light exposures and the scores of components of sleep disorder. The patients with type 2 diabetes watched TV much longer than control subjects. Their longer sedentary lifestyle such as watching TV may contribute to the development of type 2 diabetes. However, we do not know why they watched TV longer.

The chronotype and social jetlag were linked to the increase in the sleep disorder scores. The former was positively related to the INL thickness and the latter was negatively related to the ONL thickness. However, underlying mechanisms of their influence on the macular neuroretinal layer thickness were unknown.

In patients with type 2 diabetes, sleep disorder, especially obstructive sleeping apnea, is associated with diabetic peripheral neuropathy (11) and autonomic neuropathy (41). The altered median nerve amplitude, CVR-R, and beading structures of corneal nerve fibers were associated with the increased PSQI-J scores. Although we excluded the patients with confirmed diabetic peripheral neuropathy and sleep apnea, the questionable diabetic autonomic or sensory dysfunction can be a cause of sleep disorder in diabetic patients irrespective of retinal lesions. We briefly examined the influence of diabetic nephropathy on neuroretinal layer thickness and sleep disorder in the present diabetic cohort. ACR was negatively associated with the thickness at a grid of GCL, and eGFR was positively associated with the thickness at one to four grids of IPL, INL, OPL, and OPR. On the other hand, no significant association was found between scores of sleep disorder and ACR or eGFR. These results indicated that the severity of mild diabetic nephropathy did not influence sleep disorder. The association between diabetic nephropathy and neuroretinal layer thickness has to be confirmed by future studies.

In patients with type 2 diabetes, statin use improved 2 PSQI-J scores without changes in the macular neuroretinal layer thickness. The influence of statin on the sleep disorder had been inconclusive (42). Because the current study was cross-sectional and included the small number of patients using statin, prospective studies using a large number of patients with statin are needed to confirm the benefit of statin on sleep disorder.

### Strengths and Limitations

The association between sleep disorder and clinical factors, macular neuroretinal layer thickness, or temporal structures of daily life in type 2 diabetes had not been fully investigated. The novelty of the current paper is that the thinning of IPL, ONL, and photoreceptors was closely associated with sleep disorder, and the temporal structures of daily life (exposure to various light sources) influenced the thickness of the macular neuroretinal layers. As we excluded patients with clinical evidences of diabetic retinopathy and neuropathy, the influence of these complications on sleep disorder and the macular neuroretinal layer thickness seems to be minimal. Because we examined the thickness of macular neuroretinal layers in detail, the association between the individual components of sleep disorder and the thickness of specific grids of macular neuroretinal layers was obtained. Our study had some limitations. Because the sample size was relatively small and diabetic controls with diabetic neuropathy or/and diabetic retinopathy were lacking, we could not clarify the impact of these complications on sleep disorder and its association with the thickness of the macular neuroretinal layers. Because our study did not include so many morbidly obese patients (BMI > 30%: 26.6%), and excluded patients older than 55 years, we could not detect the influence of obesity and aging on sleep disorder. Therefore, we could not generalize the present results to European or American populations that include many super-obese type 2 diabetic patients. As we did not measure the arterial O_2_ concentration during sleep, the influence of hypoxia on the altered macular neuroretinal layer thickness could not be clarified. Although we found the association between the sleep disorder and the macular neuroretinal layer thinning, the causative role of the altered macular neuroretinal layers in sleep disorder remains to be clarified. Future studies are required to investigate this. The intensity of various light exposures was not evaluated, and we cannot determine the quantitative association between the amount of various light exposures and the components of sleep disorder. Finally, we did not evaluate the functions of neuroretinal layers. Although it is well-known that the ipRGCs play a key role in non-visual light sensing and regulation of circadian rhythm and sleep cycle, and signals from photoreceptors (ONL, OS) modulate the function of ipRGCs via synapse between them (IPL) (27), we cannot elucidate the influences of neuroretinal thinning on their functions relevant to impaired circadian mechanism. For elucidating the robust association between components of sleep disorder and neuroretinal alterations, the simultaneous evaluations of function and structure of neuroretinal layers are mandatory.

In conclusion, the thinning of IPL, ONL, and photoreceptors caused by hyperglycemia and dyslipidemia in type 2 diabetes without diabetic retinopathy and neuropathy was related to sleep disorder. To improve sleep disorder, controlling hyperglycemia and dyslipidemia is essential. However, interpreting the causal role of structural alterations in IPL, ONL, and photoreceptors in the sleep disorder requires a prospective study. Modifying temporal structures of daily life, especially television watching for a long time, may improve sleep disorder, and consequently, in the long term, this will improve the management of diabetes.

## Data Availability Statement

The datasets generated for this study are available on request to the corresponding author.

## Ethics Statement

Written informed consent was obtained from all subjects based on the Declaration of Helsinki. The ethics committee of Ishibashi Clinic approved the protocol of this study.

## Author Contributions

FI designed the study, researched data, and wrote the entire manuscript. MT advised on the statistical analysis, and reviewed and revised the whole manuscript. FI and MT are the guarantors of this work, and, as such, had full access to all data in the study and take responsibility for the integrity of the data and the accuracy of the data analysis and interpretation.

### Conflict of Interest

The authors declare that the research was conducted in the absence of any commercial or financial relationships that could be construed as a potential conflict of interest.

## Abbreviations

ETDRS: Early Treatment Diabetic Retinopathy Study
GCL: ganglion cell layer
INL: inner nuclear layer
IPL: inner plexiform layer
IS/OS: junction of inner and outer segment of photoreceptor
OCT: optical coherence tomography
ONL: outer nuclear layer
OPL: outer plexiform layer
OPR: outer segment of photoreceptor/retinal pigment epithelium complex
PSQI-J: Pittsburgh Sleep Quality Index Japanese Version
RNFL: retinal nerve fiber layer
RPE: retinal pigment epithelium + Bruch's membrane complex.
